# Cerebrospinal fluid drainage kinetics across the cribriform plate are reduced with aging

**DOI:** 10.1186/s12987-020-00233-0

**Published:** 2020-11-30

**Authors:** Molly Brady, Akib Rahman, Abigail Combs, Chethana Venkatraman, R. Tristan Kasper, Conor McQuaid, Wing-Chi Edmund Kwok, Ronald W. Wood, Rashid Deane

**Affiliations:** 1 Departments of Neuroscience, University of Rochester, URMC, 601 Elmwood Avenue, Rochester, NY 14642 USA; 2 Departments of Neurosurgery, University of Rochester, URMC, 601 Elmwood Avenue, Rochester, NY 14642 USA; 3 Departments of Imaging Sciences, University of Rochester, URMC, 601 Elmwood Avenue, Rochester, NY 14642 USA; 4 Departments of Obstetrics and Gynecology, Urology, University of Rochester, URMC, 601 Elmwood Avenue, Rochester, NY 14642 USA

**Keywords:** CSF, Aging brain, Interstitial fluid (ISF), CSF outflow, CSF dynamics, Nasal/across the cribriform plate CSF drainage, Spinal nerves CSF drainage, Dural CSF drainage, CSF elimination rate constants

## Abstract

**Background:**

Continuous circulation and drainage of cerebrospinal fluid (CSF) are essential for the elimination of CSF-borne metabolic products and neuronal function. While multiple CSF drainage pathways have been identified, the significance of each to normal drainage and whether there are differential changes at CSF outflow regions in the aging brain are unclear.

**Methods:**

Dynamic in vivo imaging of near infrared fluorescently-labeled albumin was used to simultaneously visualize the flow of CSF at outflow regions on the dorsal side (transcranial and -spinal) of the central nervous system. This was followed by kinetic analysis, which included the elimination rate constants for these regions. In addition, tracer distribution in ex vivo tissues were assessed, including the nasal/cribriform region, dorsal and ventral surfaces of the brain, spinal cord, cranial dura, skull base, optic and trigeminal nerves and cervical lymph nodes.

**Results:**

Based on the in vivo data, there was evidence of CSF elimination, as determined by the rate of clearance, from the nasal route across the cribriform plate and spinal subarachnoid space, but not from the dorsal dural regions. Using ex vivo tissue samples, the presence of tracer was confirmed in the cribriform area and olfactory regions, around pial blood vessels, spinal subarachnoid space, spinal cord and cervical lymph nodes but not for the dorsal dura, skull base or the other cranial nerves. Also, ex vivo tissues showed retention of tracer along brain fissures and regions associated with cisterns on the brain surfaces, but not in the brain parenchyma. Aging reduced CSF elimination across the cribriform plate but not that from the spinal SAS nor retention on the brain surfaces.

**Conclusions:**

Collectively, these data show that the main CSF outflow sites were the nasal region across the cribriform plate and from the spinal regions in mice. In young adult mice, the contribution of the nasal and cribriform route to outflow was much higher than from the spinal regions. In older mice, the contribution of the nasal route to CSF outflow was reduced significantly but not for the spinal routes. This kinetic approach may have significance in determining early changes in CSF drainage in neurological disorder, age-related cognitive decline and brain diseases.

## Background

Cerebrospinal fluid (CSF) is produced primarily by the choroid plexuses within the cerebral ventricles, and flows caudally from the lateral ventricles to the fourth ventricle before entering the subarachnoid space (SAS) in the cisterna magna, via the foramina of Luschka and Magendie [1, 2]*.* Most of the CSF then flows around the SAS of the brain and spinal cord towards multiple outflow sites and ultimately enters the systemic vascular system [1, 3, 4]. CSF outflow routes include the arachnoid villi of the dural superior sagittal sinus [5–7], olfactory nerves and across the cribriform plate into the cervical lymphatic pathway [8–11]*,* via spinal nerves into lymphatics [5, 12–16], other perineural pathways to extracranial lymphatics [17] and dural lymphatics [18–21]*.* However, the relative contribution of different outflow sites to overall CSF drainage is unknown. Furthermore, it is unclear if there are differential changes in CSF drainage at these sites with aging. To explore these major and critical gaps in knowledge to understand the role of CSF drainage in the aging brain and in brain pathology, we performed dynamic in vivo analysis at multiple drainage sites, simultaneously, on the dorsal surface of the brain and spinal column, in mice. Albumin labeled with NIRF (near infrared fluorescence) was used for in vivo imaging. In addition, albumin-NIRF and albumin labeled with Evans blue or Alexa Fluor-488 were used for ex vivo analysis of tissues. Collectively, our data show that the olfactory region across cribriform plate and the spinal regions are the main CSF outflow sites, and aging reduced the nasal/cribriform CSF outflow but not that from the spinal regions.

## Methods

### Materials

Albumin (BSA, bovine serum albumin) and Evans blue were obtained from Sigma-Aldrich, St Louis, MO). Albumin was labeled with NIRF using a kit (IRDye800CW Microscale kit, Li-COR Biosciences, Nebraska, USA) and following the manufacturer instructions. Albumin, which avidly binds to Evans blue, was labeled by incubating albumin and Evans blue (1:10 molar ratio) for 4 h, at 4 °C, and free Evans blue removed using molecular weight cut-off filter (30 kDa, Amicon Ultra Centrifugal Filter) ultracentrifugation. There was no detectable Evans blue in the filtrate. BSA Alexa Fluor-488 conjugate was obtained from ThermoFisher. ^125^I-Albumin was obtained from Perkin Elmer and used since it is regarded as a sensitive method. High energy gamma rays emitting isotopes, such as ^125^I-molecules, can be quantified relatively easily using the neat sample and radioisotopes have been used extensively as tracers in biochemical and biomedical processes, including in brain clearance studies [9, 22–25].

### Mice

C57BL/6J and Prox1Tom reporter [B6;129S-Tg (Prox1-tdTomato)12Nrud/J (Prox1Tom)] mice at 2–3 months old (Jackson Laboratory; Bar Harbor, ME, USA), and at 11–13 months (National Institute of Aging; Bethesda, Maryland) were used. All animal studies were performed in accordance with the National Institute of Health guidelines and using protocols approved by the University Committee on Animal Resources. Mice were housed in the vivarium of the University of Rochester, School of Medicine and Dentistry, on a 12:12 light/dark schedule (6AM: 6 PM) with food and water ad libitum.

### Intracisternal injections

Mice were anesthetized with isoflurane since it has fewer systemic hemodynamic effects [26]*,* and placed in their normal prone position on a temperature-controlled stage to maintain body temperature. Anesthesia was maintained with 1–2% isoflurane in oxygen. Once anesthetized, a skin incision was made in the midline from the nose to the end of the spinal cord and the skull and spine column exposed to enhance the NIRF signal intensity. Fat tissues on the back of the neck were dissected to expose the cervical spinal column. The cisterna magna was exposed, cannulated using a 30G needle, connected to a 10 µL Hamilton syringe and to a syringe pump (Infusion/Withdrawal Pump II Elite Programmable syringe pump, Harvard Apparatus, Massachusetts, USA), as reported [27]. The injection site was sealed with cyanoacrylate (All Purpose KrazyGlue, Elmer’s). Fluorescent tracers (5 μL or 0.5 μL) dissolved in aCSF, containing albumin conjugated to IRDye800CW (A-NIRF; 0.50 nmoles/μL) plus Albumin-Evans blue (Albumin-EB; 0.25%) or albumin-Alexa Fluor-488 (Albumin-AF-488; 0.25%), were intracisternally infused (0.5 μL/min) into young or older male mice. We used Albumin-EB for bright field images to confirm the association of the fluorescent tracer with CSF outflow sites and to test for labeling in other regions, such as the dura, base of skull and cranial nerves, which consistently showed no association with Albumin-NIRF in ex vivo tissues. The data show that the detection method (EB/NIRF) did not change the outcomes. The Albumin-AF-488 was used as another fluorophore to image sections of the samples.

### In vivo dynamic imaging

**Fig. 1:**
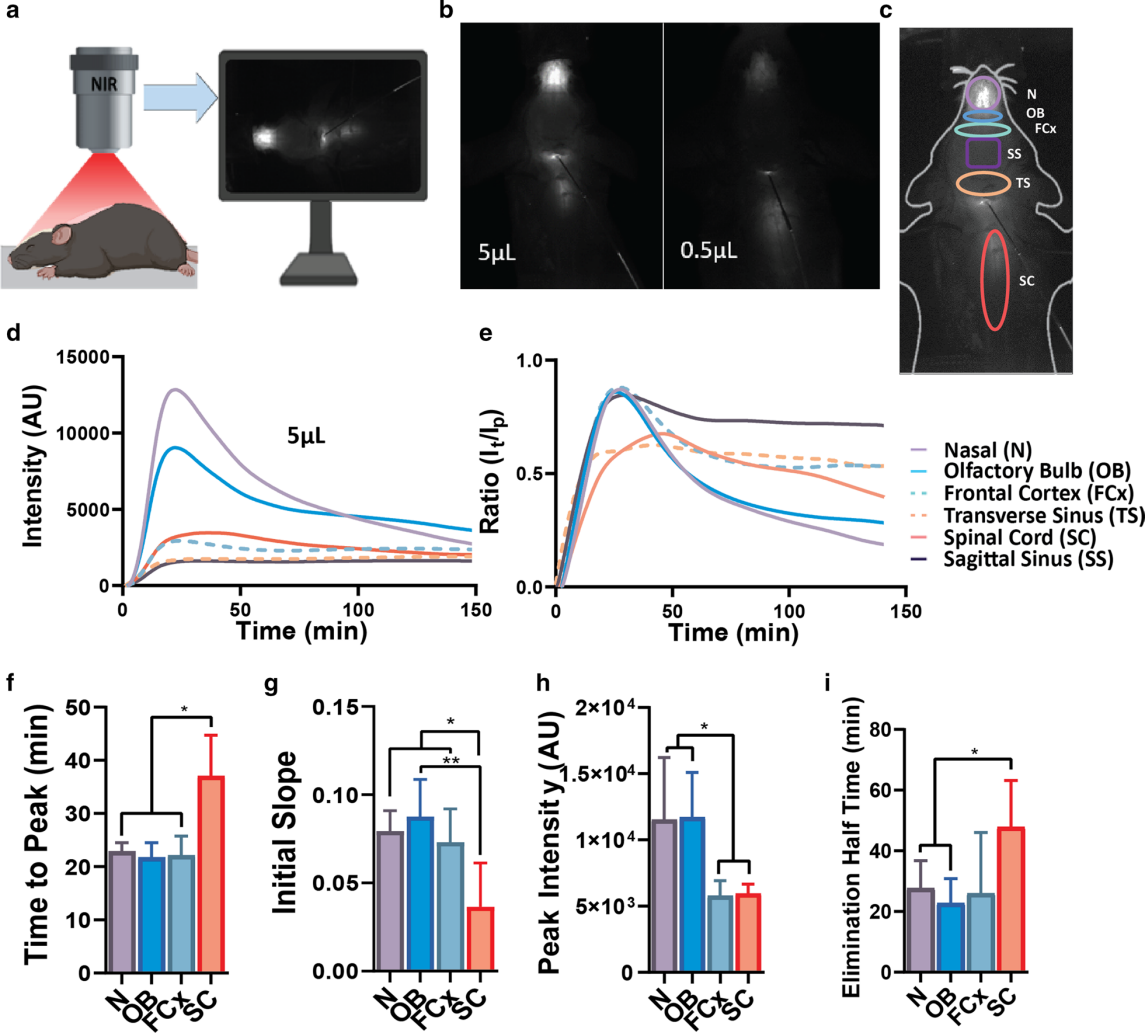
Kinetic analysis of CSF outflow shows mainly nasal and spinal routes. **a** Experimental set-up to image NIRF-Albumin in vivo in the prone mice. **b** NIRF images after 5.0 or 0.5 μL intracisternal injection at 0.5 μL/min at 30 min. **c** Regions of interest (ROIs) selected for analysis. This spinal ROI was used only at 30 min. **d** NIRF intensity-time profile for each ROI after a 5 μL intracisternal injection at 0.5 μL/min for a mouse. **e** Standardization of data by dividing intensities at each time point by the peak intensity (It/Ip ratio). Mean values were used for each ROI from 5 mice. **f** Time to reach peak intensity (Tmax) for main ROIs. **g** Rate of rise of the upward linear line using data as in panel E. **h** Peak intensities (Imax). **i** Half time (t1/2) for the falling phase (elimination) from the peak. Values are mean ± SD, N = 5 young male mice. AU (arbitrary units)

NIRF intensities were measured using a custom-made NIR system in which the NIR fluorescence was excited with light from a tungsten halogen bulb with a nondichroic, parabolic reflector to enhance NIR output, which was passed through a narrow excitation filter (Semrock, Rochester, NY, USA). The signal was measured using a Fluobeam 800 system (Fluoptics, Grenoble, France) that was composed of an electrical box containing the laser, laser power supply, an analogical/numerical module, light-emitting diode (LED) power supply and an optical head containing the charge-coupled device camera and the LED lamps. The imaging system was controlled using Fluobeam software (V3.0; Fluoptics) [28, 29]. NIRF-Albumin signal was recorded continuously (every 2 s) for up to 2.5 h from the nose to the end of the spinal column using the same imaging parameters determined by pilot experiments (Fig. 1a). Using ImageJ software (National Institutes of Health, Bethesda, MD, USA), regions of interest (ROIs) were identified, and the ROI fluorescence intensity recorded at the same settings, as described previously [28, 29]*,* using unenhanced images. The spinal column ROI was measured from the upper cervical to the upper lumbar region at 30 min (Fig. 1b, c) and from the upper cervical to the upper lumbar at 150 min (Additional file 1: Fig. S1b). The injection site was excluded from the ROI. The experimenter and the person analyzing the data were blinded to the experimental design and tracer used.

### Evaluating CSF kinetics

Using ImageJ software (National Institutes of Health, Bethesda, MD, USA), regions of interest (ROIs) were identified and defined with an appropriate-size based on the mouse skull, spine anatomy and the signal distribution from pilot experiments (Fig. 1c; Additional file 1: Fig.S1b). The ROI fluorescence intensity was recorded at the same settings using unenhanced images, as described previously [28, 29]. The spinal column ROI was measured from the upper cervical to the upper thoracic region at 30 min (Fig. 1b, c) and from the upper cervical to the upper lumbar at 150 min (Additional file 1: Fig. S1b). The injection site was excluded from the ROI. Kinetic analysis was performed at 150 min. Each ROI was imaged over the duration of the experiment to capture all detectable signals. Mean pixel intensity within each ROI was quantified for each time point using the same parameters and images without enhancement. Average intensity of the first ten seconds for each ROI was used as the background and subtracted from intensity at each time point. Similarly, peak intensity (Imax) was identified as the average of 10 s at the peak identified using GraphPad Prism. Time to peak (Tmax) was calculated using the time from injection to the maximum intensity value. Initial slopes were calculated by applying a linear regression to the rising phase of the ROI intensity profile using the increase in signal for the upward linear part of this phase. To correct for experimental variation, the intensity at each time point was divided by peak intensity to standardize the data. The area under the curve was determined by performing area under the curve analysis for the standardized data in GraphPad Prism software. The half time was determined from the falling phase of the ROI intensities (from the peak to the trough intensity) using the exponential function in the GraphPad Prism software.

### Ex vivo imaging of whole tissues

At the end of in vivo imaging, animals were sacrificed and tissue samples harvested including whole brain, skull cap (dorsal dura), skull base (ventral dura), cervical lymph nodes, spinal column and spinal cord. Brain was used to analyze tracer intensity at the dorsal and ventral surfaces and within brain parenchyma, skull cap for tracer at the dural venous sinuses and lymphatic vessels (almost the same areas), skull base for tracer distribution, spinal column for the tracer distribution within the spinal SAS and possible drainage sites, spinal cord for association of the tracer at the surface and cervical lymph nodes for lymphatic drainage as a possible drainage site for the CSF tracer. Tissues were washed equally and imaged individually with the same parameters used for in vivo imaging. ImageJ software was used to measure intensities without enhancement. Evans blue bright field image was acquired using a conventional camera.

### Tissue preparation for fluorescence imaging

After the duration of the experiment, mice were transcardially perfused using cold phosphate buffered saline (PBS) and paraformaldehyde (PFA) (4% in PBS, pH: 7.3). Tissues samples were removed, stored overnight in PFA at 4 °C, re-stored in cold PBS and cut into 100 µm coronal sections using a Vibratome (Leica VT1000E). Lymph nodes were embedded in agarose gel before sectioning. Spinal columns were cut into cervical, thoracic, and lumbar regions, embedded in Optimal Cutting Temperature (OCT) compound and cut into 40 μm sections using a cryostat. One section for every 500 µm was mounted on Superfrost Plus glass slides using ProLong Gold Antifade Mountant medium (ThermoFisher Scientific; Waltham, MA, USA) for fluorescence imaging (VS120 Virtual Slide Microscope, Olympus). Exposure and gain were fixed for all experimental groups based on pilot experiments.

### Plasma albumin

To determine CSF transfer into blood, tracer levels of ^125^I-Albumin (0.05 μCi; PerkinElmer Inc. MA, USA), were intracisternally injected at 0.5 μL/min for 10 min (total volume 5 μL) and after 30 min blood samples collected by cardiac puncture in heparinized tubes. Blood was centrifuged to separate plasma, which was collected and counted for radioactivity using a gamma counter (Wallac Vizard Gamma Counter; PerkinElmer).

### Statistical analysis

Data were analyzed by analysis of variance (ANOVA) followed by post hoc Tukey test using GraphPad Prism 8.0.2 software. The differences were considered to be significant at *p* < 0.05. For statistical representation, *P < 0.05, **P < 0.01, and ***P < 0.001 and ****P< 0.0001 are the levels of statistical significance. NS is not significant. All values were expressed as mean ± SD. Student’s *t*-test with Welch’s correction was used for two samples. GraphPad Prism software was used for all analysis including kinetic analysis and area under the curve (AUC).

## Results

### The predominant routes of CSF drainage are across the cribriform plate and via the spinal subarachnoid space.

Albumin (66 kDa), the most abundant protein in CSF, distributes mainly with CSF flow (e.g., [10]). Since we cannot track endogenous albumin, we used labeled BSA to analyze real-time in vivo CSF drainage kinetics of a macromolecule at multiple sites under the same experimental conditions. BSA has been used extensively to study CSF and interstitial fluid (ISF) distribution [7–11, 30]. In addition, NIRF, which avoids the natural background fluorescence of biomolecules, and thus, provides a better contrast between the target and background, has been used with minimal invasive methods (e.g., [28, 29]). To establish a suitable injected volume 0.5 and 5 μL were used in pilot experiments at 30 min time point. The intensities from the brain and spinal column were greater (fivefold to sevenfold) for the larger than that of the smaller volume, as expected (Fig. 1a, b). The larger volume was selected to obtain better signals at all regions of interest (ROIs). In an additional test, NIRF-Albumin signal from under an ex vivo control skull-cap was recorded with the camera above the skull-cap under the same conditions used for in vivo recordings (Additional file 1: Fig. S1a). Conjugated albumin (5 µl) was intracisternally infused (0.5 μL/min) into anesthetized young mice (2–3 monthsold) and NIRF-Albumin signal recorded continuously (every 2 s) for up to 2.5 h from the nose to the end of the spinal column with mice in a flat prone position (Fig. 1a). Signals were recorded in the predetermined ROIs: nasal area and across the cribriform plate (N), olfactory bulb (OB), frontal cortex (FCx), sagittal sinus (SS), transverse sinus (TS) and spinal column (SC; Fig. 1c). The spinal column ROI was measured from the upper cervical to the upper thoracic region over the duration of the experiment without enhancement at 150 min (Additional file 1: Fig. S1b). The FCx was included as it is near the OB and olfactofrontal cistern and had significant signal even though it is not a known CSF drainage site. Peak NIRF-Albumin signals were greater for the N and OB regions and decreased exponentially within 2.0 h (Fig. 1d), the turnover time for mouse CSF [1]. In contrast the signals for the dural sinuses (SS and TS) remained almost constant after an initial small rise, indicating little CSF elimination through the dural routes compared to the nasal route (Fig. 1d). The spinal column region, after an initial rise, showed a slower falling phase compared to that for the nasal regions (Fig. 1d). Data were standardized to the peak signal to correct for experimental variations, and the same patterns for the signal profile at the ROIs were observed (Fig. 1e). Consequently, we focused on N, OB, FCx, and on the SC region. The peak times (Tmax) were not significantly different for the N, OB and FCx regions. Tmax was 1.7-fold longer for the SC than that of the N, OB, or FCx regions (Fig. 1f), suggesting a more rapid CSF flow to the N, OB regions. To test this, the rate of rise of the NIRF-Albumin signal (linear regression of the upward slope) was determined. This was 2.2-fold slower for the SC compared to the nasal regions, which were similar (Fig. 1g). Thus, the rate of CSF flow to the SC was slower than that to the other regions and the peak intensities (Imax) were similar for the N/OB regions, but were 1.9-fold higher than for the SC (Fig. 1h). Additional file 1: Table S1 shows the data for Tmax, slope and Imax for the SS and TS regions. Prior work in rats, injected intracisternally with 80 μL of gadolinium-immunoglobulin G (Gd-IgG), also estimated peak times from MRI but it is difficult to directly compare their results [31] with those obtained here.

The Imax is dependent on both the rate of CSF delivery to the ROI and the rate of elimination. The calculated half-time clearance (t1/2) for CSF elimination was 1.6-fold faster from the nasal route compared to that of the SC (Fig. 1i). The area under the curve (AUC), the overall regional NIRF-Albumin exposure, was increased by 1.4-fold for the SC and the frontal cortex compared to the nasal regions (Additional file 1: Fig. S1c). Collectively, these data show that the main CSF drainage pathways when assessed simultaneously after intracisternal injection were via the olfactory region across the cribriform plate and spinal subarachnoid space (possibly via the spinal nerves) but not via the dural routes. A recent report came to same conclusion even with an injection of 20 μL at 2 μL/min into each lateral ventricle of contrast agent, in rats [32]. Earlier reports have shown the nasal and across the cribriform plate route [7, 9–11, 24, 33, 34] or spinal route/spinal nerves [5, 13, 17] for macromolecules and small molecules, in various animals and using different methods. Unlike earlier studies, this kinetic analysis showed that there was a faster CSF flow from the cisterna magna to the nasal region with faster elimination compared to the SC. This could explain the sharper peak and lower AUC for the nasal region compared to that of the SC. To determine whether the intracisternal injection volume alters the CSF drainage, the same kinetic experiments were performed with 0.5 μL but at the same injection rate and concentration. There was no significant change to the elimination kinetics (Additional file 1: Fig. S1d). Surprisingly, there was no evidence of CSF elimination (falling phase of the intensity vs time profile curve) from the dura (SS and TS) compared to the nasal and spinal regions.

### Ex vivo analysis: regions of CSF drainage

**Fig. 2:**
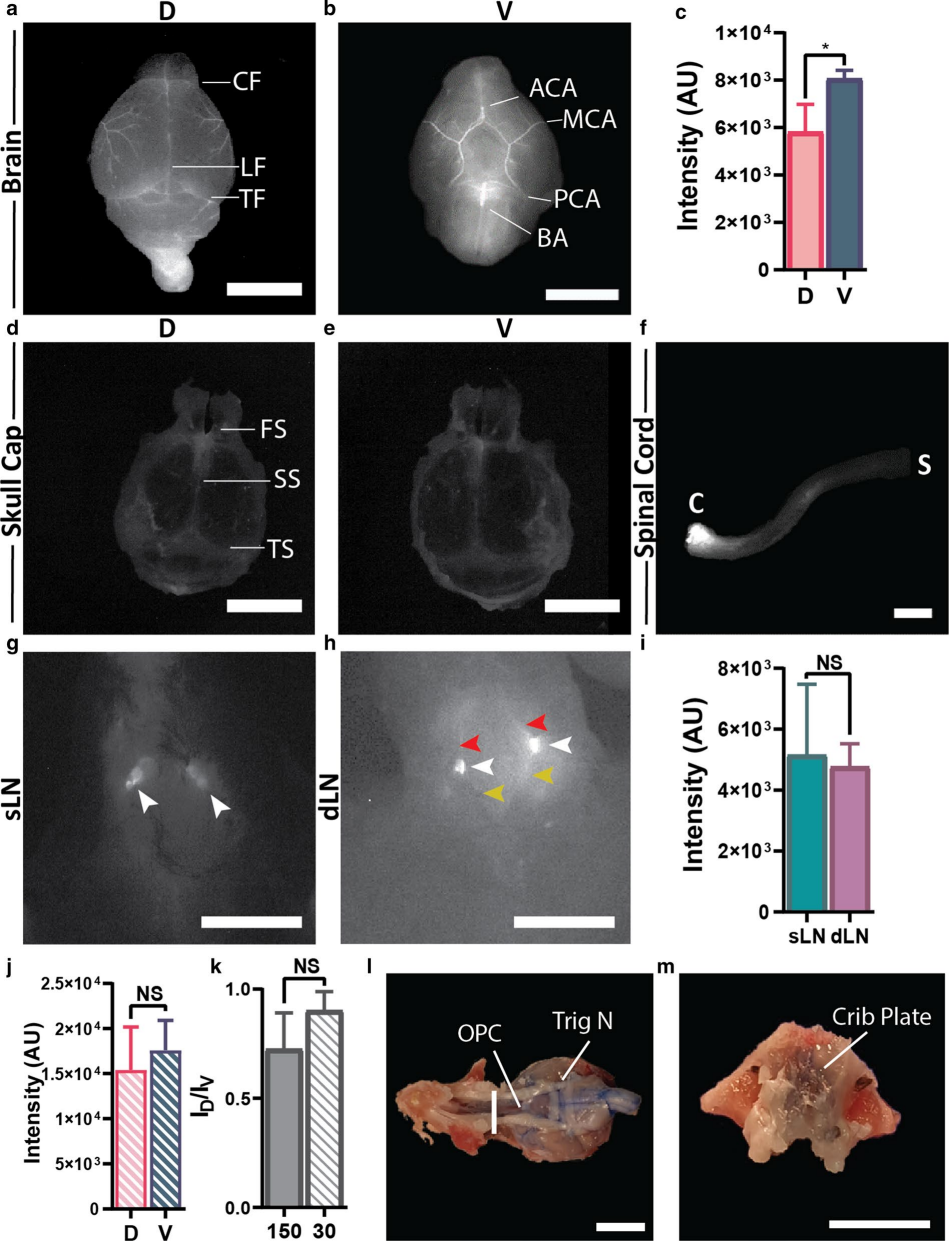
Ex vivo images of CSF outflow regions show mainly nasal and spinal routes. Representative images of the dorsal **a** and ventral **b** brain surfaces revealed retention of tracer along blood vessels, fissures and brain surfaces after 150 min. **c** Quantification of intensities on the dorsal and ventral surface using the same imaging and analysis parameters as in the in vivo studies. Representative images of the dorsal **d** and ventral **e** skull cap surfaces with intact dura, spinal cord (**f;** C (cervical) and S (sacral)), and superficial (sLN; **g**) and deep (dLN; **h**) cervical lymph nodes (white arrow heads) and afferent and efferent lymphatic vessels (red arrow heads) in the exposed neck region. **i** Quantification of NIRF intensities in the sLN and dLN. NIRF images were enhanced equally to show all possible locations of NIRF-Albumin after 150 min. CF (circular fissure), LF (longitudinal fissure), TF (transverse fissure), ACA (anterior cerebral artery), MCA (middle cerebral artery), PCA (posterior cerebral artery), BA (basilar artery), FS (frontonasal suture), SS (sagittal sinus), TS (transverse). **j** Quantification of intensity for the dorsal and ventral brain surfaces 30 min post-injection. **k** Comparison of dorsal to ventral intensity ratios at 30 and 150 min. Representative Albumin-Evans blue (EB) bright field images after 30 min for the ventral brain surface with intact skull cap (**l**) and the cut surface across around the cribriform plate region (**m**) at the white line in panel L. OPC (optic nerve chiasm), Trig N (trigeminal nerve) and Crib P (cribriform plate area). Residual pigment from the eyes is shown at the top and residual EB at the bottom. Values are mean ± SD, N = 5 young male mice. Scale bar: 5 mm

Since there was NIRF-Albumin signal for the in vivo dural sites (SS and TS) recorded trans-cranially but little CSF elimination from these sites, tissue samples were removed at the end of the experiment (150 min) and imaged ex vivo. To better visualize the location of NIRF-Albumin, the images were enhanced equally for comparison. The dorsal and ventral brain surfaces after removal of the dura plus skull (skull cap), showed the presence of NIRF-Albumin, especially along the major arterial vessels (anterior, middle and posterior cerebral, circle of Willis and basilar) and in the fissures: longitudinal (under the SS), transverse (under the TS), and circular (under the olfactofrontal cistern) (Fig. 2a, b), compared to non-injected brains (Additional file 1: Fig. S2a). However, NIRF-Albumin intensity (quantified by using the unenhanced images) on the ventral brain surface was 1.4-fold greater than that of the dorsal surface (Fig. 2c), which indicates that CSF flow occurred around the brain with a significantly greater ventral flow and/or greater retention. In contrast, there was little NIRF signal for SS and TS regions of the intact dura on the surface of the skull-cap when imaged on the dorsal (Fig. 2d) or ventral surfaces (Fig. 2e) compared to non-injected mice (Additional file 1: Fig. S2b). Signal at the frontonasal suture (above the olfactofrontal cistern) area may represent residual NIRF-Albumin from the OB/circular fissure areas and olfactofrontal cistern, which had high NIRF intensities. While the brain surfaces without enhancement showed signal there was little for the dura (Additional file 1: Fig. S2c, d). The skull base (ventral dura) shows no significant signal even with enhancement except for the injection site (Additional file 1: Fig. S2e). There was signal associated the spinal cord posterior to the injection site within the ROI (Fig. 2f), but not at the caudal end of the enhanced image. Thus, confirming that there was no detectable tracer flowing out of the ROI at the caudal end into the sacral region. The superficial and deep cervical lymph nodes were associated with the tracer (Fig. 2g, h) but there were no significant differences between intensities for these lymph nodes (Fig. 2i). Thus, this could represent CSF from the SAS entering the cervical lymphatics as a CSF outflow pathway to blood, as demonstrated earlier for decades (e.g., [52]). For example, BSA injected into the lateral ventricles was collected from the efferent lymphatic vessels and from the cervical lymph nodes e.g., [8, 10]. Since the NIRF-Albumin signal at the ROIs was decreased by 150 min post-injection due to CSF flow and drainage, some experiments were performed after 30 min, around the peak time. NIRF-Albumin signals were similarly associated with the blood vessels and fissures on the ventral and dorsal brain surfaces and the spinal cord but not for the dura (Additional file 1: Fig. S3a–d). However, at 30 min the intensities for the dorsal and ventral brain surfaces were 2.3-fold higher (Fig. 2j) compared to that at 150 min (Fig. 2c), which reflects a greater NIRF-Albumin delivery at the peak time. However, there was no significant difference between the dorsal and ventral intensities at 30 min (Fig. 2j), nor for the dorsal to ventral intensity ratios between these time points (Fig. 2k). This suggests that CSF is distributed around the brain at about the peak time, but there was retention (ventral > dorsal surface), perhaps, in the fissures and around vessels, after 150 min but not at 30 min. These findings were confirmed with Albumin-Evans blue (EB) bright field images, which showed EB was associated with the arteries, fissures, spinal cord and cervical lymph nodes but not with the intact dura (Additional file 1: Fig. S3e–k). Albumin-EB was present within the area of the cribriform plate (Fig. 2l-m) when compared to non-injected mice (Additional file 1: Fig. S3l). There was no significant association of the tracer with the optic or trigeminal nerves (Additional file 1: Fig. S4a–d). There was some tracer close to the spinal nerve routes (Additional file 1: Fig. S4e, f). However, further work is needed to confirm these observations. Collectively, these images confirmed that the nasal, via the cribriform plate, and spinal routes, via the spinal SAS, are the main CSF drainage pathways.

### Ex vivo analysis of tissue sections using Albumin Alexa Fluor -488

**Fig. 3:**
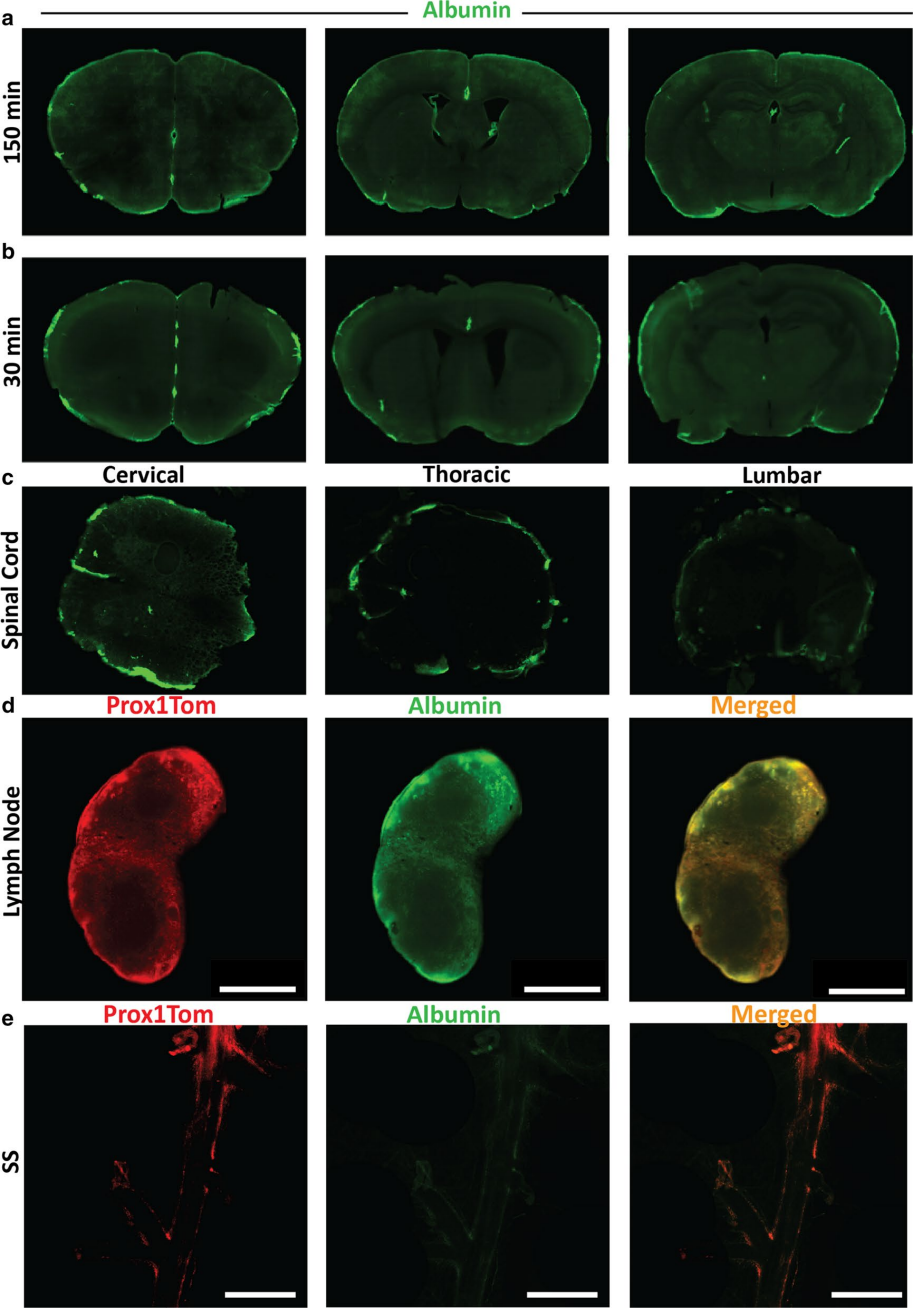
Albumin tissue distribution at CSF outflow regions. Representative images of Albumin-Alexa Fluor-488 in frozen sections show no significant parenchyma distribution at 150 min (**a**) or at 30 min (**b**). Each section is 500 μm apart. **c** Representative images of Albumin-Alexa Fluor-488 distribution for the spinal column (cervical, thoracic and lumbar regions) at 150 min. **d** Prox1Tom expression (lymphatic vessels) in the superficial cervical lymph node, (red), Albumin-Alexa Fluor-488 distribution (green) and merged images at 30 min showing no significant levels of the tracer. **e** Images of the dura showing Prox1Tom expression (red), Albumin-Alexa Fluor-488 distribution (green) and merged images at 30 min. There is no significant levels of the tracer associated with Prox1 Tom (lymphatic vessels). N = 3 young male mice. Scale bar: 5 mm

To establish whether the albumin tracer from cisternal CSF enters brain parenchyma, samples were sectioned and imaged. While there was Albumin-488 around the circumference of brain sections and major pial vessels, there was no significant signal within the parenchyma at either150 or 30 min (Fig. 3a, b), compared to autofluorescence in non-injected brains (Additional file 1: Fig. S5a). Thus, there was little diffusion or fast flow into the brain parenchyma, as reported for India ink [11] and for sucrose, a small molecule [35]. In addition, Albumin-488 fluorescence signal was mainly confined within the SAS of the spinal cord (Fig. 3c), and within the cervical lymph nodes or lymph vessels (Additional file 1: Fig. S5b, c). To confirm the role of lymphatic vessels, Prox1Tom lymphatic vessel reporter mice were used. There was Albumin-488 fluorescence signal in the cervical lymph nodes, which colocalized with lymphatic vessels (Fig. 3d). The presence of Prox1Tom-positive vessels was confirmed in the dura in the transgenic mice (Additional file 1: Fig. S5d), but there were no significant levels of albumin with these vessels or any vessels (Fig. 3e). These images confirm that the SAS is the main pathway for CSF drainage from the cisterna magna, but with no significant/little flow into the brain parenchyma or dural regions compared to that at the nasal/across the cribriform plate and spinal regions. It also indicates that transcranial recording or brain uptake studies after intracisternal injection of tracers likely includes significant tracer retention on the brain surface, in the subarachnoid CSF and the cisterns.

### Aging differentially alters CSF drainage kinetics

**Fig. 4:**
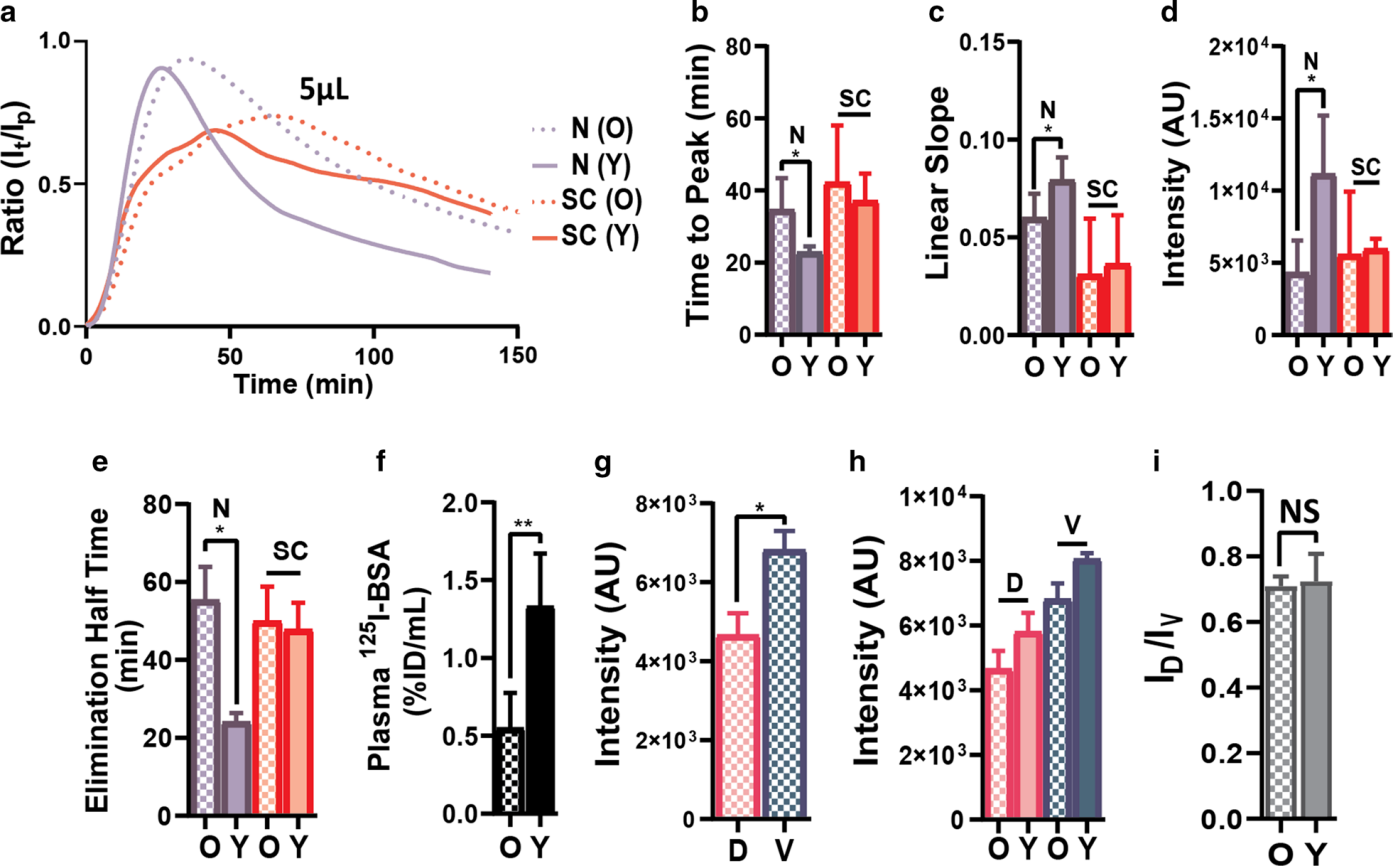
Aging alters the CSF outflow kinetics at the nasal route. **a** NIRF intensity-time profile at the main CSF outflow regions for young (Y) and old (O) mice after 5 μL intracisternal injection at 0.5 μL/min. **b** Time to reach peak intensity (Tmax) for nasal (N) and spinal cord (SC) regions. (**c**) Rate of rise of the upward linear slope using data as in panel A. **d** Peak intensities (Imax). **e** Half time (t1/2) for the elimination phase. **f** Plasma ^125^I-BSA levels after intracisternal injection. **g** NIRF-Albumin intensity for the dorsal and ventral brain surfaces after 150 min in the old mice. **h** Comparison of dorsal and ventral brain surfaces intensities between young and old mice. **i** Comparison of dorsal to ventral brain surface intensities ratios between young and old mice. Values are mean ± SD, N = 5–6 mice per group. Young male mice (2–3 months--old). Old male mice (11–13 months-old). AU (arbitrary units)

**Fig. 5:**
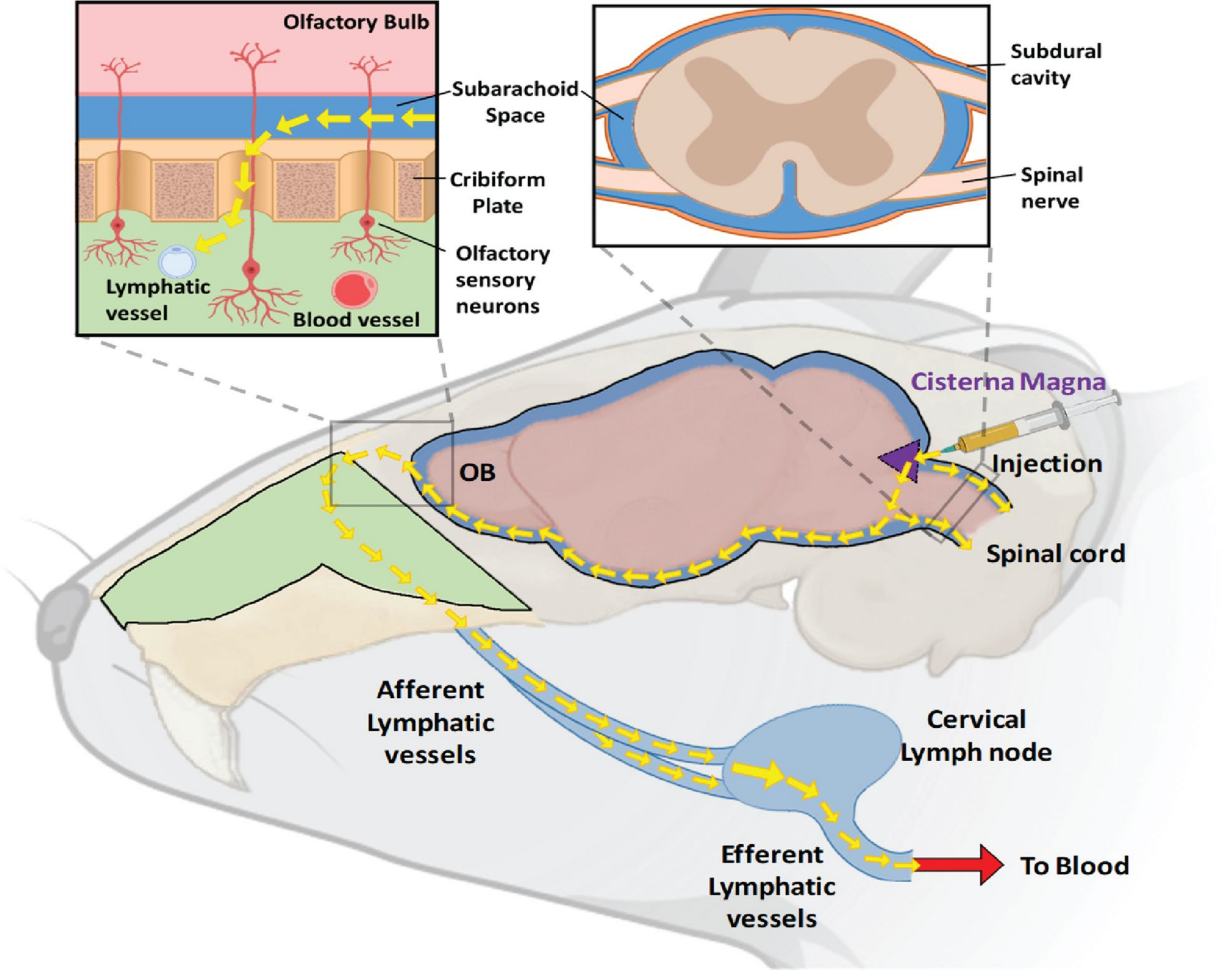
Schematic diagram illustrating the main CSF outflow pathways and working model. *OB* olfactory bulb

Older mice (11to13 monthsold) were used to determine whether age affects the elimination kinetics of albumin tracer from the CSF. The general pattern of the intensity profile curves for the ROIs was similar to that of the younger mice (Additional file 1: Fig. S6a). Since the nasal and spinal routes were the main outflow pathways, we focused on these regions for comparison. The intensity profiles for the N and SC were changed in the older mice compared to their younger counter part (Fig. 4a). In the older mice, the peak time was 1.4-fold later for the N regions compared to the younger mice, but there was no significant change for the SC (Fig. 4b). While OB and FCx followed the nasal/across the cribriform plate region pattern there was no significant change with age for SS and TS (Additional file 1: Fig. S6b). The initial slope was reduced in the older mice for the N by 1.3-fold, while there was no significant change for the SC (Fig. 4c). Also, the slopes were decreased for the other ROIs in the older mice compared to the younger mice (Additional file 1: Fig. S6c). In young mice, the nasal peak intensity was greater, by 2.7-fold, compared to the old mice without significant changes in the SC (Fig. 4d). Similarly, the half time for elimination phase in the older mice for the N region was 2.3-fold longer compared to that of the younger mice with no significant changes for the SC (Fig. 4e). The AUC was significantly greater in the older mice by 1.6-fold for the N region but not for the SC compared to the younger mice (Additional file 1: Fig. S6d). The plasma level of intracisternal injected ^125^I-albumin was reduced by 1.4-fold in the older mice compared to that of the younger ones (Fig. 4f), suggesting that CSF outflow into blood was reduced with aging. In the older mice, NIRF-Albumin signals were present on the brain surfaces and SC but not at the dura (Additional file 1: Fig. S6e–h). While there was a lower NIRF-albumin intensity for the brain surfaces in the older mice, like the younger mice, this was greater on ventral surface (Fig. 4g), but there were no significant differences in intensities between the dorsal or ventral surfaces between the young and old groups (Fig. 4 h) nor for the dorsal to ventral intensity ratios between the two ages (Fig. 4i). Thus, aging differentially affects CSF drainage kinetics by reducing the delivery to and elimination from the main nasal/across the cribriform plate outflow region but not significantly changing that at the spinal regions. In addition, the data show that brain surface retention was not significantly altered by aging, which may reflect some non-specific retention in all the grooves on the brain surfaces. Using the half time to estimate the contribution of these two main CSF elimination routes, indicates that in the older mice the nasal/cribriform plate route contribution is about 0.8-fold that of the spinal, as aging affected mainly nasal drainage kinetics (Fig. 4g). This may reflect the reduced CSF secretion seen in aging animals and humans [17, 36–41]. Figure 5 shows a schematic diagram of the main CSF flow pathways in mice and working model.

## Discussion

In these studies, we did not introduce any new CSF drainage sites, terminologies, tracers, methodologies or quantitative analysis (elimination rate constants) to the field. In addition, we did not study ISF clearance. Instead, we determined clearance of tracers from the CSF in vivo by measuring simultaneously the elimination rates over 150 min at potential drainage sites on the dorsal surface of the mouse’s brain, and spinal column. Since our in vivo imaging cannot see the ventral surfaces of the brain and spinal cord nor the skull base (ventral dura) we also performed ex vivo imaging to assess association of the tracer with additional potential drainage sites, which included the optic and trigeminal nerves and skull base. Collectively, our data (both in vivo and ex vivo) show that the nasal tissue and across the cribriform plate and the spinal regions are the main CSF drainage sites. Elimination from the nasal and cribriform region, but not that from the spinal sites, was reduced in aging mice.

We hypothesized, based on published data, that CSF would be eliminated via multiple routes including the dural outflow sites (venous sinuses and dural lymphatics located in the same area). Thus, we were surprised that the dural sites were not significant drainage routes compared to that of the nasal and spinal routes. We used an imaging system that preferentially images the dorsal dura as the NIRF camera was above the skull and spinal column.

We showed that there was a faster distribution of NIRF-Albumin from the cisternal CSF to the nasal regions, which attained a higher maximum intensity followed by a faster elimination rate, compared to that of the spinal routes. Ex vivo tissue images confirmed that the SAS is the main pathway for CSF drainage from the cisterna magna. There was no significant fast CSF inflow into the brain parenchyma, as reported for glymphatics (e.g., [42]). We showed that tracers are retained on the brain surfaces (dorsal and ventral), which were associated with blood vessels, fissures and cisterns, with significant retention of NIRF-Albumin signal even after 150 min. While there was no significant difference of tracer levels between the dorsal and ventral brain surface at 30 min, there was a difference at 150 min where ventral was greater than dorsal. Despite this distribution, there was no significant association of the tracer with the dura (dorsal and ventral (skull base) or the optic and trigeminal nerves. There appeared to be no significant CSF elimination from the SAS to the dura, compared to that at the nasal and spinal regions. Recent reports have inferred CSF drainage from the SAS to dural lymphatics by showing association of tracer (e.g., Evans blue) with the dorsal dura [18, 19, 43]*.*

### Nasal and spinal routes as the main CSF elimination regions

Our data confirm earlier studies that macromolecule tracers in CSF flow from the SAS to the olfactory bulb, through the cribriform plate leading to the nasal submucosa and according to previous studies, ultimately to the cervical lymphatic vessels and nodes, and from the spinal SAS leading to spinal CSF outflow pathways, such as spinal nerves and lymphatics [7–11, 30, 33, 44–53]. Thus, our finding on the CSF drainage sites are similar to previous studies using a number of tracers, such as India ink, radiolabeled albumin and a range of molecules of different size, to track CSF flow (e.g., [7, 10, 47]). CSF outflow from the cranium has been shown to occur within sheaths of cranial nerves, such as the olfactory nerve to peripheral lymphatic vessels that flow into lymph nodes, such as cervical lymph nodes [10, 11, 34, 54–56]. CSF from the spinal SAS may flow along spinal nerves [5, 12] through the spinal arachnoid villi [49, 57–59], along spinal nerve roots to epidural lymphatics [46] and spinal meninges to dural lymphatics [15]. We now show that the elimination rate of CSF from the nasal/cribriform plate region is greater than that from the spinal region in mice, but that this is slower in aging mice. In addition, we have shown that under the conditions of these experiments there is no significant CSF elimination from the dura, the optic and trigeminal nerves or the skull base compared to that at the nasal and spinal regions, since ex vivo analysis showed that the optic and trigeminal nerves and the skull base were not associated with the tracer. Thus, these regions may not be significant CSF drainage sites compared to the nasal and spinal routes within the time frame of these experiments.

The entire spinal column was in the field of view, and the ROI was placed where tracer was detected for the duration of the experiment. The fixed spinal ROI was set-up to accommodate pulsatile (to and forth) flow (e.g., [60–65]). At the rostral end of the ROI, we excluded the injection site, the source of tracer to flow in all directions. At this end, outside the ROI, tracer signal was not significantly increased even at 30 min (Fig. 1b, c). In these experiments, tracer levels within the ROI increased progressively to a maximum after about 25% of the total experimental duration (150 min). Outside the caudal end of the ROI, there was no detectable tracer signal at the end of the experiment. Also, there was no detectable tracer signal at the caudal end of the spine (sacral end) in the ex in vivo spinal cord, even with enhancement (Fig. 2f). Thus, there is no evidence of significant tracer flow out of the ROI. If there was a flow out of the spinal ROI then the pattern of the intensity- time profile would look different from our data. Assuming the resistance to flow, intrathoracic and intraabdominal pressures and cardiovascular-related oscillations are unchanged then the volume of flow per unit time should be constant. If there was no flow due to pulsation then the tracer intensity should be almost the same within the ROI. In our studies, signal intensity increased within the ROI to a peak, indicating that there is a net flow into the ROI which then falls exponentially. There is tracer’s distribution within the ROI as evident from the width of the intensity-time plot. Albumin diffusion coefficient is 9.35 × 10^–7^ cm^2^/s i.e., 0.008 cm^2^/150 min, at 37 °C [66], so this cannot account for the tracer flow on its own. The disappearance of regional intensity reflects elimination at all spinal drainage sites that have been suggested in previous studies (e.g., see [1, 3]). How CSF is eliminated from the spinal SAS is not completely clear. So far, we have not seen any signal in the sacral lymph nodes or any other lymph nodes except in the neck region. This may be related to the chosen injection site, cisterna magna; previous studies have shown signal in sacral lymph nodes after injecting into the lateral ventricle [15]. Further studies are needed to confirm the actual sites of spinal CSF drainage, and the role of the sacral lymph nodes in this process.

We did not see any tracer associated with the optic and trigeminal nerves or the dura (dorsal and ventral (skull base), but saw tracer in the nasal/cribriform and spinal regions, major pial blood vessels, and brain surfaces of ex vivo samples. We also confirmed these results using Evans blue-albumin. Tracer has been seen previously to be associated with the trigeminal nerve and skull base [21, 31]. Previous studies have shown that the optic nerve was associated with tracer after intraparenchymal injection (ISF clearance) but this was not seen in all experiments [67]. Indeed, the frequency of tracer association with the olfactory bulb was greater than that of the optic nerve. Our study is different in that we injected into the CSF (cisterna magna) and studied CSF elimination. While CSF and ISF elimination may share some common pathways, this is not always the case: following ISF injections in the caudate putamen (a common site used in these studies) about 15 to 30% of labeled albumin entered the CSF [9, 24, 68]. How tracer is associated with the optic and trigeminal nerves or the dura (both dorsal and ventral) is unclear. It is possible that the faster flow across the cribriform plate and posterior along the spinal SAS may limit association of the tracer with these nerves and the dura. It is also possible that tracer in these routes might flow at a much slower rate due to a high tissue resistance compared to the nasal and spinal regions. The permeability of these nerves to different tracers in the CSF is also unclear. It is possible that there is a molecular weight cut-off for the tracer uptake into the optic nerve [69] or a specific region for uptake [16]. It is also unclear if the physio-chemical properties of the tracer, its concentration and experimental design affect tracer’s association with these nerves and the dura. Further work is needed on CSF elimination sites from the dura, skull base and the optic and trigeminal nerves.

### Dural lymphatics in CSF elimination

In fluid balance, the lymphatic system collects excess tissue fluid due to an imbalance in the filtration/reabsorption process at the capillaries, and returns it to blood. The tight junctions between cerebral endothelial cells (blood brain barrier, BBB) and low permeability for polar molecules across the endothelium accounts for the low hydraulic conductivity of the cerebrovasculature [1, 70, 71]. Unlike peripheral tissues, there are no lymphatic vessels in the CNS parenchyma (e.g., [1, 3]). The dura lacks BBB characteristics, but like peripheral tissues, has lymphatic vessels (e.g., [18, 75]). Thus, it may perform similar functions as peripheral tissue lymphatics. In contrast, the arachnoid is part of the CNS barrier system (e.g., [75]). It appears that there is no pathway for subarachnoid CSF to directly access the dural lymphatics due to the arachnoid membrane barrier, although SAS CSF can enter the dural venous sinuses and return to blood directly (e.g., [1]). Our data do not support the suggestion that the dura has a significant role in CSF elimination, in mice, as reported (e.g., [18, 20, 21, 43]). This discrepancy may be due to different experimental conditions used in these studies [30]. If the dural routes (blood or lymphatic) are involved in CSF elimination from the SAS, in mice, then it is extremely slow compared to the nasal and spinal route, and undetectable in our experiments. Our data are in accord with earlier quantitative approaches, which have shown using different molecules of various molecular weights, that the olfactory/cribriform plate/cervical lymphatic is a significant CSF drainage pathway [10, 24, 67, 73, 74]* and* from more recent reports [17, 31, 32].

### CSF inflow into brain Parenchyma

We found no significant CSF flow from the SAS into the brain parenchyma, as reported previously [30], in contrast to glymphatic CSF inflow along arterial paravascular space (e.g., [42]). We used an infusion rate (0.5 μL/min) just outside the range of CSF secretion rate (0.3–0.4 μL/min) in mice [1], and a similar volume (5 μL), to that previously used (e.g., [27, 75]). This volume is about 14% of the estimated CSF volume (35 μL) in mice [1]. Using these parameters, we did not see any significant influx of BSA from the SAS into the parenchyma. In our earlier studies, we used 5 μL at 1 μL/min and showed some inflow of tracer in CSF from the SAS to brain parenchyma [27, 75]. A greater parenchymal inflow from SAS was seen with 10 μL at 2 μL/min (e.g., [42]) in mice, and for 80 μL at 1.6 μL/min in rats [31, 76, 77]. In young rats, the CSF secretion is about 2 μL/min and the CSF volume is about 200 μL [32] so the the injection regimen in rats was close to the CSF secretion rate, but the injected volume was about 40% of the CSF volume. Injection into the CSF and/or brain may cause an increase in intracranial pressure at the site of injection and throughout the cranium, and the magnitude and duration of the increase depends on the rate of injection and on volume injected (e.g., [48]). To this aim we may have achieved a method that minimized the inflow of CSF tracer from the SAS into the brain parenchyma.

Our data confirm the present of CSF tracers alongside major pial vessels, presumably the perivascular compartment, as described previously (e.g., [11, 67, 72]). However, there was no significant uptake in the parenchyma even after 150 min, as reported earlier (e.g., [11, 35, 67]). This is likely due to a pulsation flow along the perivascular compartment that may limit fast entry into the parenchyma [78, 79]. In our studies the retention of tracer was confined to the brain surfaces, and this was similar for the dorsal and ventral brain surfaces at 30 min (about the peak time). At 150 min, the ventral surface had a greater intensity. Recent studies showed a greater ventral brain distribution compared to that of the dorsal, but this included parenchymal uptake [31]. Thus, using our extracranial recordings of CSF tracers to study brain uptake also detected tracer located on the brain surface, retention in fissures, in the pial perivascular compartment and CSF in the subarachnoid space.

### Aging and CSF drainage

We show that aging differentially affects the CSF drainage kinetics by reducing delivery to and elimination from the main nasal/cribriform plate outflow site but not significantly changing that for the spinal regions. Using elimination t1/2 to estimate the contribution of these two main routes to the outflow of CSF indicates that the nasal route contributes 1.5 times that of the spinal in young adult mice, and aging reduced the nasal contribution to 0.8 that of the spinal (Fig. 4e). Using the AUC for NIRF-Albumin at the nasal and spinal regions, the two main routes for CSF outflow, to estimate the proportion of CSF drainage indicates that nasal/cribriform plate contributes 60% and spinal 40%, in young mice (Fig. 4d). This is similar to the ratio (75:25) reported previously [82]. It is likely that the proportion of CSF flowing via the nasal and spinal routes may depend on experimental conditions [48, 80, 81]. Our studies were performed under isoflurane anesthesia, the mice were in their normal prone position, and a low rate of injection and low injected volume were used to minimize injection-induced flow disturbances. There was no significant change to CSF elimination between 0.5 and 5 μL injected into the cisterna magna; a larger pool of CSF may also contribute to minimizing injection-induced flow changes.

In the older mice, the contribution of the two main CSF elimination regions was about the same. Aging has been shown to reduce the rate of CSF secretion and this may lead to a reduction in drainage [38–41] and CSF drainage into the lymphatics [17]. Aging did not affect the association of tracer with the brain surfaces. With aging, the route with higher CSF outflow (nasal/cribriform plate) deteriorates more quickly than the spinal route. Thus, therapies to increase CSF outflow in the aging brain may delay the onset of CSF-related neurological disorders in the brain aging and age-related decline in cognition.

### Limitations of our approach

The primary objective was to determine the elimination rate of tracer in CSF at already-known CSF outflow sites. A major limitation was that our in vivo imaging system cannot assess sites on the ventral brain surface, such as the optic and trigeminal nerves, and the skull base. We used additional ex vivo tissues to look for the association of CSF tracer with those regions previously identified as outflow sites, especially on the ventral brain surface and the skull base. At the end of the 150 min there was some retention of tracer at all the sites imaged in vivo. CSF may be trapped in the various fissures and cisterns in the SAS and remain there until it is eliminated at the CSF outflow sites and a longer imaging time could produce a different result. A further limitation is that the exact location of tracer cannot be distinguished. For example, the longitudinal fissure, which separates the two hemispheres, is under the superior sagittal sinus and dural lymphatics so transcranial recording for the SS also includes signals from this fissure, brain surface blood vessels, subarachnoid CSF. This limitation is also similar for the recordings from the nasal and spinal regions, in that tracer signals come from the subarachnoid CSF, brain, or other surrounding tissues. We used minimal invasive surgery hence our imaging system could not identify the specific spinal drainage sites. Further work is needed to confirm the spinal CSF drainage sites.

## Conclusions

In mice, using CSF labeled albumin, the nasal regions across the cribriform plate and spinal regions were the main CSF elimination sites using both in vivo and ex vivo data within the conditions of these experiments. There was no significant CSF outflow from the SAS to the cranial dura. Tracer was associated with the nasal/cribriform and spinal regions but not with the optic and trigeminal nerves or the skull base. Also, there was significant retention of NIRF-Albumin on the brain surfaces, including in fissures and around pial vessels. There was no significant inflow from the SAS into the brain parenchyma. In young adult mice, there was a faster CSF elimination rate from the nasal/cribriform plate compared to the spinal regions. For the first time, we show that aging differentially affects the CSF drainage kinetics by reducing delivery to and elimination from the nasal/cribriform plate outflow site but not significantly changing that at the spinal regions. Thus, delayed CSF outflow could be associated with the onset of brain aging, neurodegeneration and age-related decline in cognition.

## Supplementary Information

**Additional file 1: Figure S1. **Analysis of CSF outflow kinetics. **Figure S2.** Ex vivo images of CSF outflow regions at 150 min. **Figure S3.** Ex vivo images of CSF outflow regions at 30 mins. **Figure S4.** Images of the optic nerves and ventral spinal column at 30 min. **Figure S5.** Albumin distribution at the cervical lymph nodes and Prox1Tom dural expression. **Figure S6.** Aging alters CSF outflow kinetics. **Table S1.** Deducible kinetic parameters for the superior sagittal sinus (SS) and transverse sinus (TS).

## Abbreviations

aCSF: Artificial cerebrospinal fluid
AUC: Area under the curve
CSF: Cerebrospinal fluid
EB: Evans blue
FCx: Frontal cortex
MRI: Magnetic resonance imaging
N: Nasal
NIRF: Near infrared fluorescence
OB: Olfactory bulb
ROIs: Regions of interest
SAS: Subarachnoid space
SC: Spinal cord
SS: Sagittal sinus
TS: Transverse sinus
