# Supplementary material for: Cerebrospinal fluid drainage kinetics across the cribriform plate are reduced with aging
Source: Fluids Barriers CNS. 2020 Nov 30;17:71. doi: 10.1186/s12987-020-00233-0 (PMC7706057; doi:10.1186/s12987-020-00233-0)
Supplement: Supplementary file 1 — Additional file 1: Figure S1. Analysis of CSF outflow kinetics. Figure S2. Ex vivo images of CSF outflow regions at 150 min. Figure S3. Ex vivo images of CSF outflow regions at 30 mins. Figure S4. Images of the optic nerves and ventral spinal column at 30 min. Figure S5. Albumin distribution at the cervical lymph nodes and Prox1Tom dural expression. Figure S6. Aging alters CSF outflow kinetics. Table S1. Deducible kinetic parameters for the superior sagittal sinus (SS) and transverse sinus (TS). [file 12987_2020_233_MOESM1_ESM.pdf]

Title: Cerebrospinal fluid drainage kinetics across the cribriform plate are reduced with aging

Authors: Molly Brady[1], Akib Rahman[1], Abigail Combs[1], Chethana Venkatraman[1], R. Tristan Kasper[2], Conor McQuaid[1], Wing-Chi Edmund Kwok[3], Ronald W Wood[1,4], Rashid Deane[1,2]*

Departments of Neuroscience[1], Neurosurgery[2], Imaging Sciences[3], Obstetrics and Gynecology[4], Urology[4], University of Rochester, URMC, 601 Elmwood Avenue, Rochester, NY 14642.

*Correspondence to: *Rashid Deane. Email address: *Rashid_Deane@urmc.Rochester.eduXxx

Supplementary Fig. S1

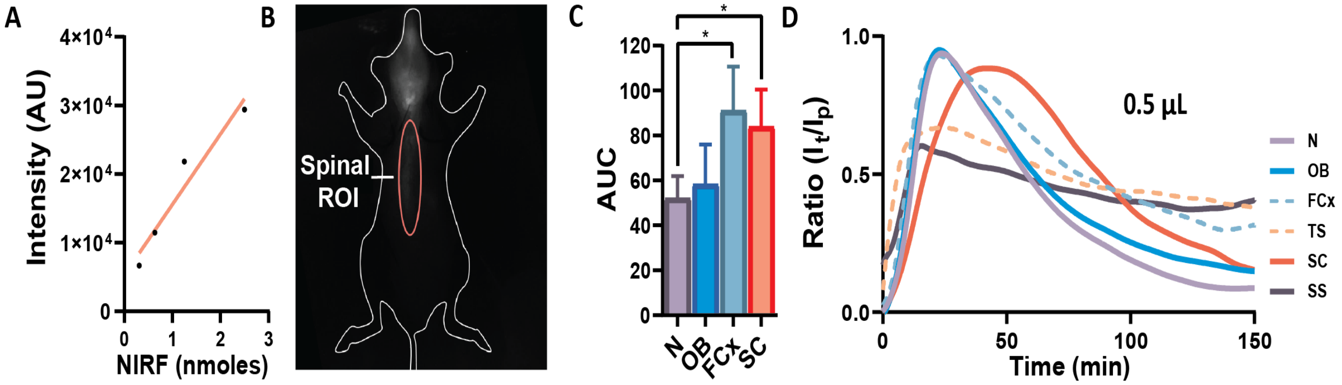

Supplementary Fig. S1. Analysis of CSF outflow kinetics.

(**A**) NIRF intensities at different levels of NIRF-albumin placed in an Eppendorf tube and under an isolated control skull cap. **B**) Spinal regions of interest (ROIs) selected for analysis at 150 mins based on detectable tracer signal (from the upper cervical to the upper lumbar) and the imaging of the entire spine. (**C**) Area under the curve (AUC) for the 5 µL intracisternal injection. (**D**) Intensity profile at each ROI after 0.5 µL intracisternal injection at 0.5 µL/min. Values are mean ± SD, N=5 young male mice. AU (arbitrary units). N (nasal), OB (olfactory bulb), FCx (frontal cortex), TS (transverse sinus), SC (spinal cord) and SS (superior sagittal sinus).

Supplementary Fig. S2

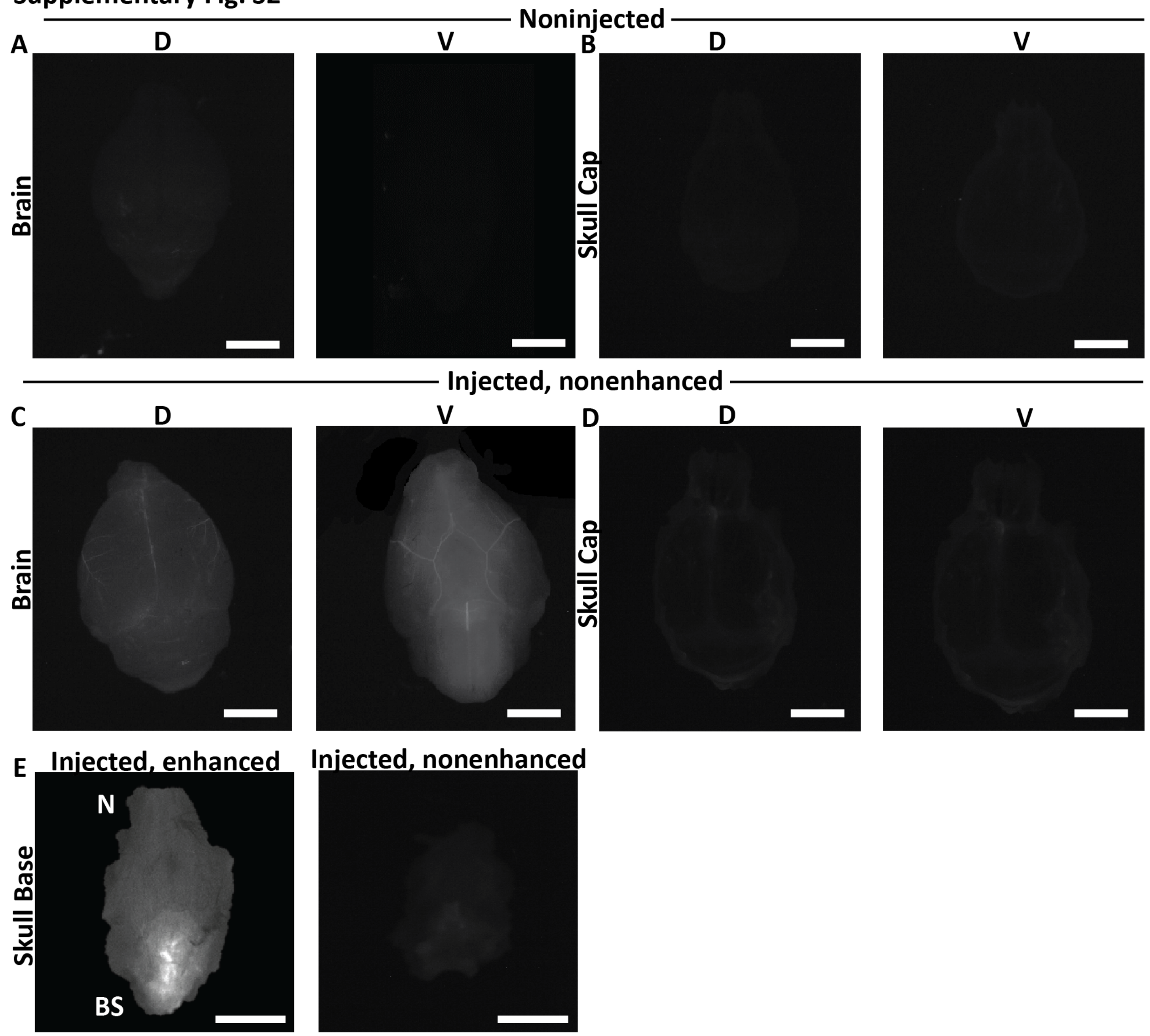

Supplementary Fig. S2. Ex vivo images of CSF outflow regions at 150 min. Representative images of the dorsal (D) and ventral (V) brain surfaces (**A**) and the dorsal and ventral skull cap (**B**) of the non-injected control mice. (**C-D**) Representative non-enhanced images of the dorsal and ventral brain surfaces (**C**) and the dorsal and ventral skull cap (**D**). Representative images of the skull base under the brain after enhancement (left) and non-enhanced (right). N=5 young male mice. N (nose), BS (brain stem). Scale bar: 5 mm.

Supplementary Fig. S3

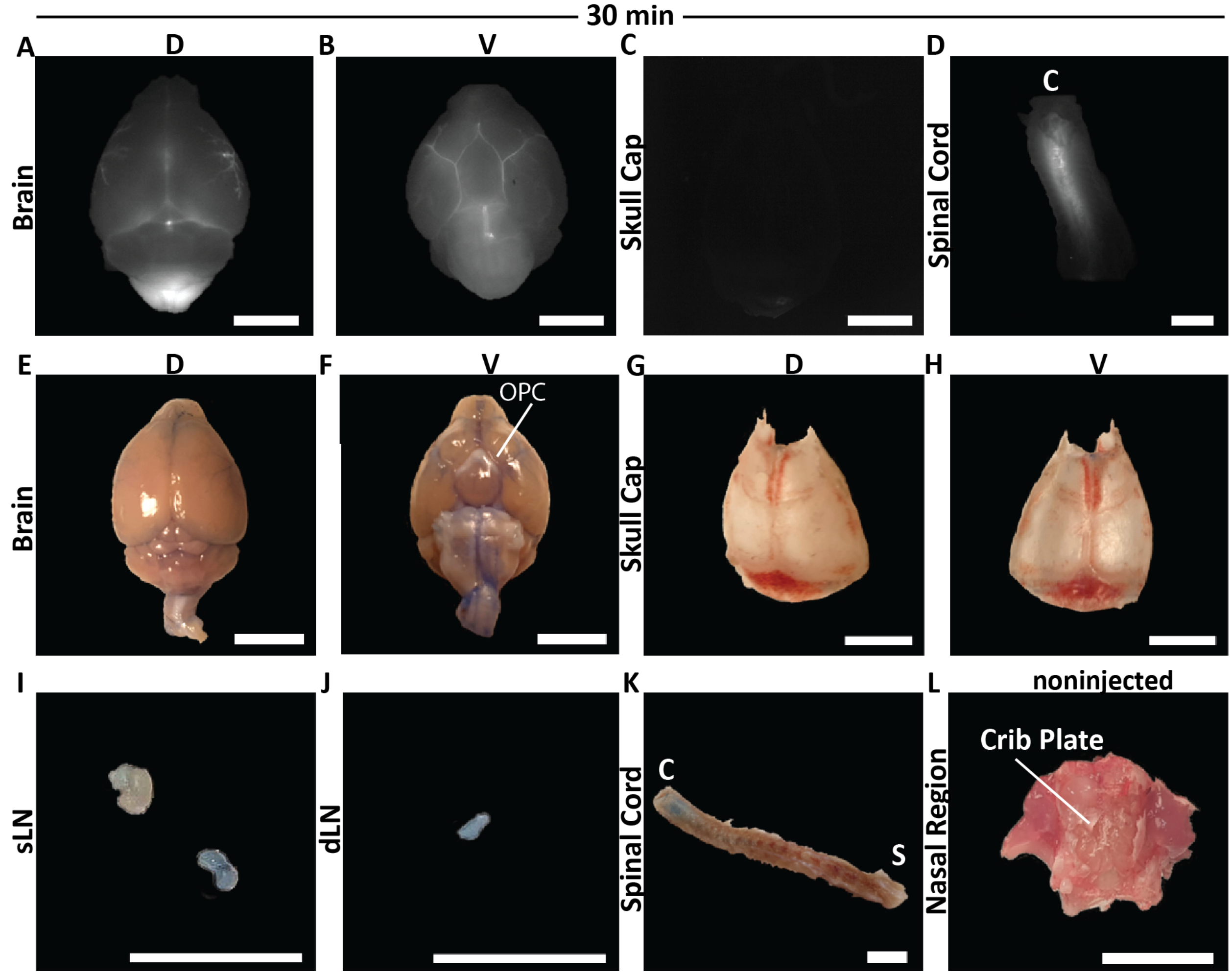

Supplementary Fig. S3. Ex vivo images of CSF outflow regions at 30 mins.

(**A-K**) Representative images for the dorsal (**A**) and ventral (**B**) brain surfaces, skull cap with intact dura (**C**), dorsal spinal column surface (**D**) without enhancement. (**E-K**) Representative Albumin-Evans blue brightfield images of the dorsal (**E**) and ventral (**F)** brain surfaces, dorsal (**G**) and ventral (**H**) skull cap with intact dura, superficial cervical lymph nodes (**I**) and deep cervical lymph node (**J**) and spinal column (**K**). (**L**) The cut surface across the cribriform plate (Crib Plate) region of a control non-injected mouse. C (cervical), S (sacral) N=5 young male mice. Scale bar:5 mm

Supplementary Fig. S4

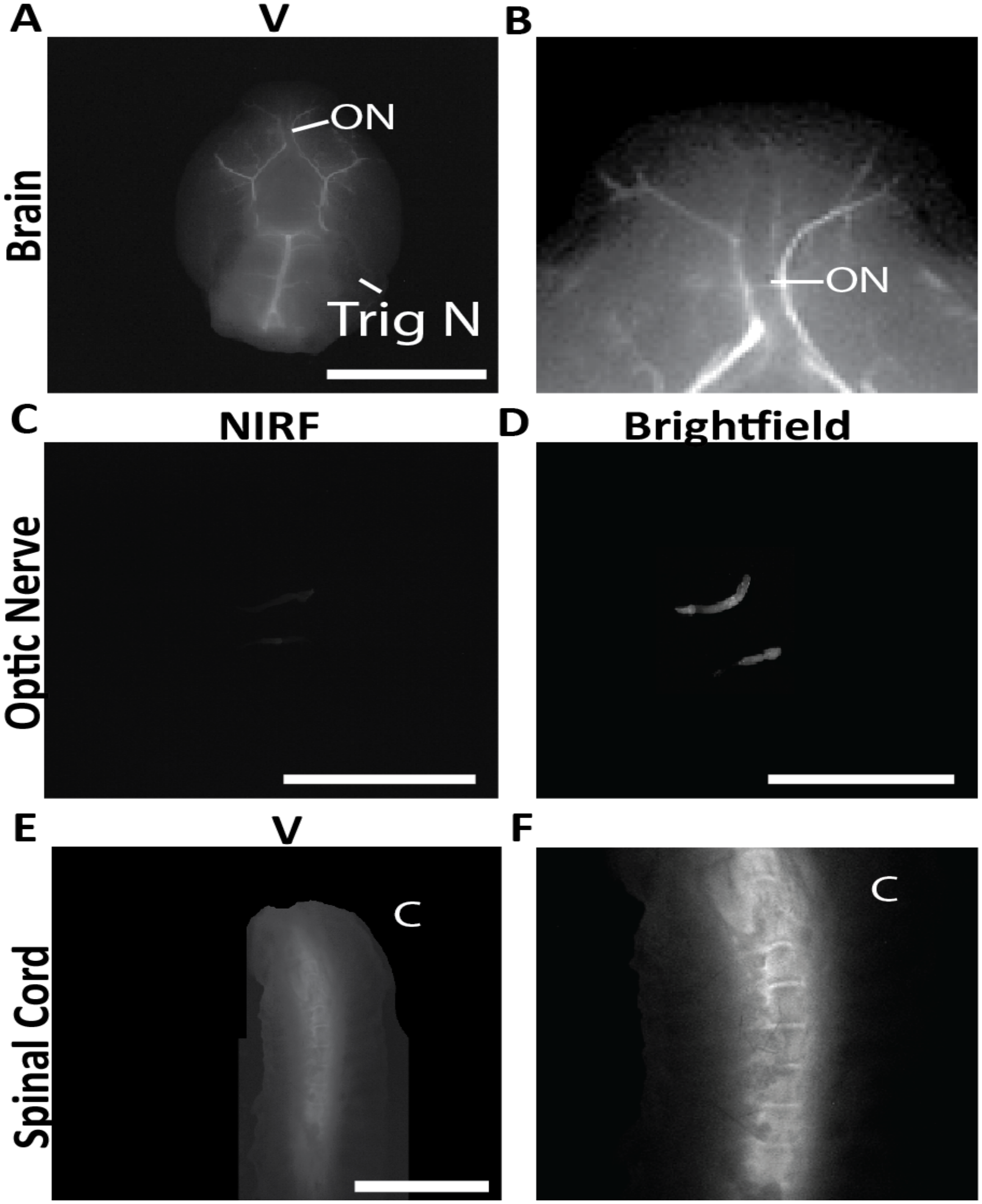

Supplementary Fig. S4. images of the optic nerves and ventral spinal column at 30 min. Images of the ventral (V) brain surfaces (**A-B**) showing the optic (ON) and trigeminal (Trig N) nerves were not significantly labeled with the tracer. **B**) Magnified image of the ON region from panel A. NIRF (**C**) and bright field (**D**) images of the isolated ON. (**E-F**) Images of the spine's

ventral surfaces. **F**) Magnified image of panel E, showing possible CSF outflow via spinal nerves. C (cervical spinal region). N=5 young male mice. Scale bar (5 mm).

Supplementary Fig. S5

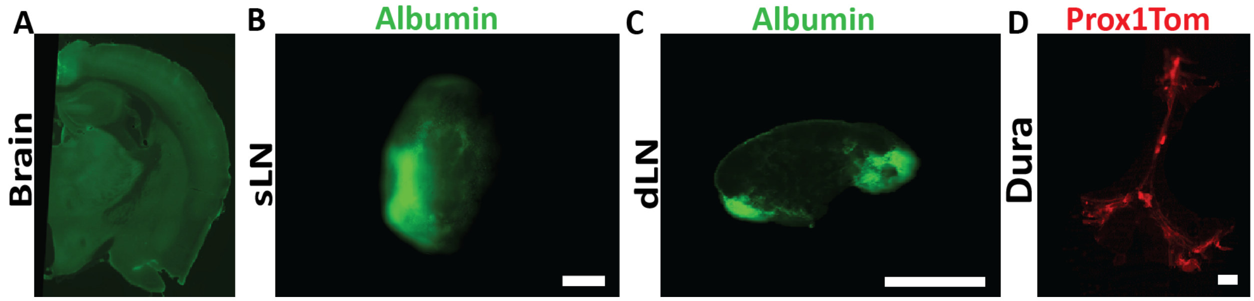

Supplementary Fig. S5. Albumin distribution at the cervical lymph nodes and Prox1Tom dural expression. A) Representative image (autofluorescence) in the green channel of control brain without any Alexa Fluor-488 injection. **B-C**) Representative images of Albumin-Alexa Fluor-488 distribution at 150 min in the superficial lymph node (sLN) and deep lymph node (dLN; green). **D**) Representative images of Prox1Tom (lymphatic vessels) expression in the dura of control non-injected transgenic mice (red), showing lymphatic vessels, N=3 young male mice. Scale bar:5 mm.

Supplementary Fig. S6

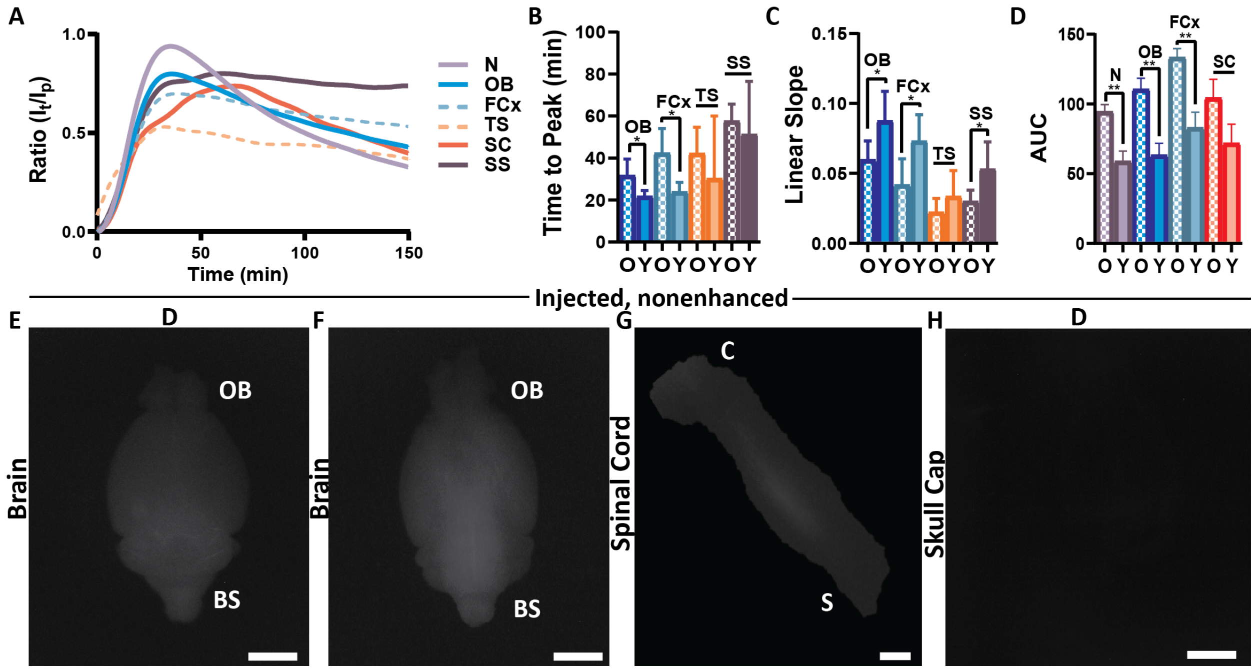

Supplementary Fig. S6. Aging alters CSF outflow kinetics.

(**A**) NIRF-Albumin intensity-time profile at regions of interest for old (O) mice after 5 μL intracisternal injection at 0.5 μL/min. (**B**) Time to reach peak intensity (Tmax) for the ROIs, olfactory bulb (OB), frontal cortex (FCx), transverse sinus (TS) and sagittal sinus (SS) comparing young (Y) and old (O) mice. (**C**) Rate of rise of the upward linear slope for the OB, FCx, TS and SS comparing young (Y) and old (O) mice. (**D**) Area under the curve (AUC) for N, OB, FCx and SC comparing young (Y and old (O) mice. Representative NIRF images of the dorsal (**E**) and ventral (**F**) brain surfaces, spinal cord (**G**) and skull cap (**H**) for the old mice at 150 min without enhancement. BS (brain stem, C (cervical), S (sacral). Values are mean ± SD, N=5-6 mice per group. Scale bar: 5 mm. AU (arbitrary units)

Supplementary Table S1: Deducible kinetic parameters for the superior sagittal sinus (SS) and transverse sinus (TS)

	TS	SS
Tmax	30.25 ± 19.83	66.13 ± 39.67
Slope	0.062 ± .0594	0.053±0.01951
Imax	8339 ± 3979	3803 ± 508.8

Mean ± SD, n=5
